# Increased intrathecal neurofilament light and immunoglobulin M predict severe disability in relapsing-remitting multiple sclerosis

**DOI:** 10.3389/fimmu.2022.967953

**Published:** 2022-08-10

**Authors:** Igal Rosenstein, Sofia Rasch, Markus Axelsson, Lenka Novakova, Kaj Blennow, Henrik Zetterberg, Jan Lycke

**Affiliations:** ^1^ Department of Clinical Neuroscience, Institute of Neuroscience and Physiology at Sahlgrenska Academy, University of Gothenburg, Gothenburg, Sweden; ^2^ Clinical Neurochemistry Laboratory, Sahlgrenska University Hospital, Mölndal, Sweden; ^3^ Department of Psychiatry and Neurochemistry, Institute of Neuroscience and Physiology, University of Gothenburg, Mölndal, Sweden; ^4^ UK Dementia Research Institute at University College London (UCL), London, United Kingdom; ^5^ Department of Neurodegenerative Disease, University College London (UCL) Queen Square Institute of Neurology, London, United Kingdom; ^6^ Hong Kong Centre for Neurodegenerative Diseases, Hong Kong, Hong Kong SAR, China

**Keywords:** multiple sclerosis, cerebrospinal fluid, neurofilament light, intrathecal IgM synthesis, biomarkers, prognosis

## Abstract

**Background:**

Emerging evidence supports that determination of intrathecal immunoglobulin M (IgM) synthesis (ITMS) and neurofilament light (NfL) concentration in cerebrospinal fluid (CSF) may be clinically useful as disease severity biomarkers in relapsing-remitting multiple sclerosis (RRMS).

**Methods:**

Monocentric observational longitudinal cohort study in which prospectively collected data were retrospectively retrieved. Included were patients with RRMS (n=457) who had a diagnostic investigation including analysis of ITMS and CSF neurofilament light (cNfL). ITMS was calculated with the linear index formula, the intrathecal fraction of IgM according to Reiber (IgM_IF_), and by qualitative determination of oligoclonal IgM bands (OCMB). Univariable and multivariable models were performed to predict Evidence of Disease Activity-3 (EDA-3) status within 24 months from onset, and the risk of Expanded Disability Status Score (EDSS) ≥3 and ≥6.

**Results:**

All investigated methods to calculate ITMS significantly predicted evidence of disease activity (EDA-3) within 24 months. IgM_IF_>0% showed the strongest association with EDA-3 status (adjusted hazard ratio [aHR] 3.7, 95%CI 2.7-5, p<0.001). Combining IgM-index>0.1 or OCMB with increased cNfL were strong predictors of EDSS≥3 (for cNfL**^+^**/IgM-index**^+^**: aHR 4.6, 95%CI 2.6-8.2, p<0.001) and EDSS≥6 (aHR 8.2, 95%CI 2.3-30, p<0.001).

**Conclusions:**

In a real-world setting, ITMS was a useful biomarker in early RRMS to predict disabling MS and its prognostic value was even stronger in combination with cNfL. Our data suggest that determination of ITMS and cNfL should be included in the diagnostic work-up of RRMS for prognostic purposes and in decisions of disease-modifying therapy.

## Introduction

Multiple sclerosis (MS) is an inflammatory and degenerative disease of the central nervous system (CNS). The clinical course of MS is heterogeneous, including patients with benign course with essentially preserved functions to those with highly aggressive course (1), often characterized by frequent relapses, high magnetic resonance imaging (MRI) lesion burden, and rapid development of significant disability (2). Early intervention with highly efficacious disease modifying therapies (DMTs) may halt inflammatory activity and reduce the risk for severe disability (3, 4). Several clinical and MRI measures that may reflect the severity of the disease are considered for treatment decisions. However, there is a need to explore early and easily available prognostic biomarkers to identify patients who are at greater risk to develop aggressive and disabling MS disease.

Accumulating data on the role of diagnostic intrathecal IgM synthesis (ITMS) suggest that it may serve as a marker of an aggressive MS course and a recent literature review summarized the majority of these studies and concluded that there is strong evidence that ITMS is a negative prognostic marker (5). Numerous studies have previously demonstrated that ITMS in patients with clinically isolated syndrome (CIS) have increased risk to convert to clinically definitive MS (CDMS) (6–13). However, results from other studies are conflicting, including some with large study populations (14–18).

The presence of oligoclonal IgG bands (OCGB) in CSF indicates dissemination in time according to the 2017 revised McDonald criteria (19), and there is some evidence that clonal intrathecal IgG synthesis (ITGS) predicts severity of MS (17). However, studies that directly compared ITGS with ITMS with regards to prediction of disease severity have shown that ITMS is more reliable (12, 20). Over the years, various methods to quantify the presence of ITMS have been developed (5), including the linear IgM-index, the intrathecal fraction of IgM (IgM_IF_) according to Reiber’s formula (10, 12, 17), and qualitative detection of oligoclonal IgM bands (OCMB) (18, 21), as well as lipid-specific OCMB (11, 22). However, it remains to be determined which of these methods most reliably estimates ITMS.

Other cerebrospinal fluid (CSF) biomarkers previously have shown valuable prognostic information. Glial fibrillary acidic protein (GFAP), a biomarker of astrogliosis (23) has been associated with progression (24, 25). Chitinase 3-like protein 1 (CHI3L1) and chemokine (C-X-C motif) ligand 13 (CXCL13) may both reflect inflammatory disease activity but also show prognostic value (18, 26). The most promising CSF biomarker to predict clinical worsening is neurofilament light (cNfL) (27, 28), a biomarker of axonal injury. However, contrary to measures of intrathecal immunoglobulin synthesis, they are rarely included in the diagnostic work-up of MS and the predictive value of combining some of these biomarkers is unknown.

The aim of this study was to validate ITMS in a real world setting as a predictor of a more aggressive disease course as determined by rapid disability progression (29–31) and to investigate whether combining ITMS and cNfL, biomarkers reflecting different pathophysiological processes in RRMS, may improve their prognostic capacity.

## Material and methods

### Study design and study population

**Figure 1:**
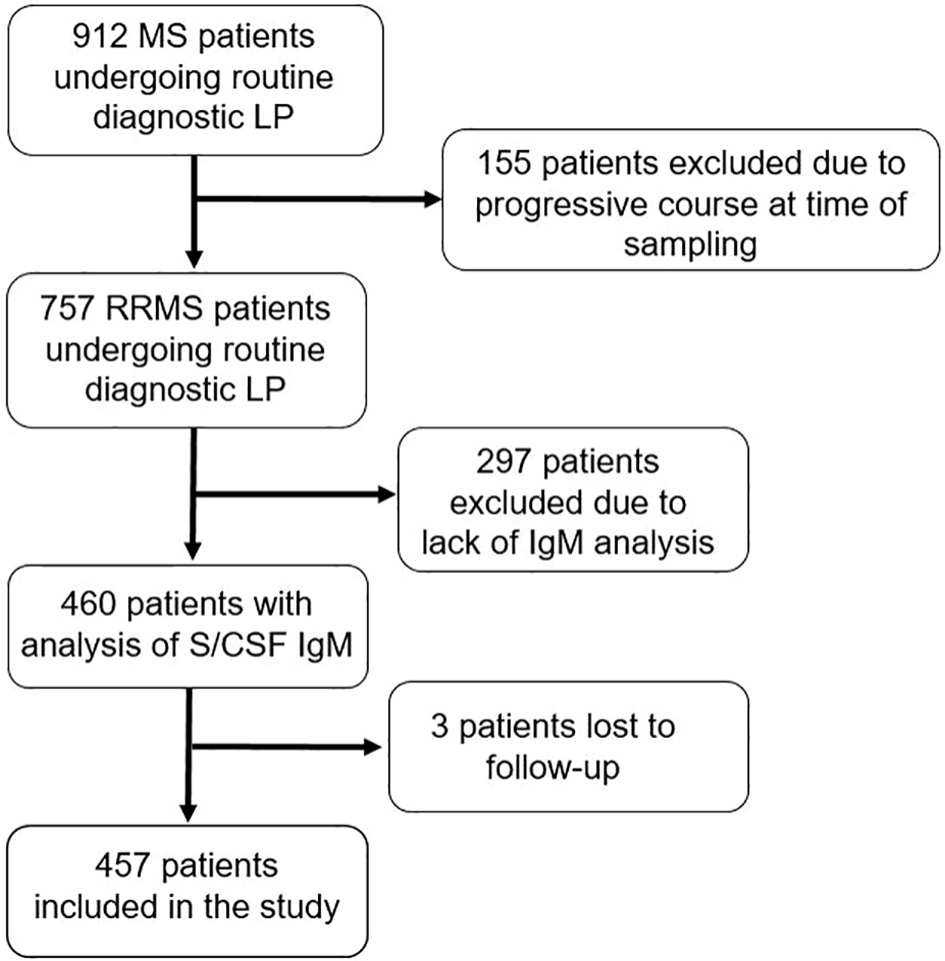
Flow chart of the selection of patients with relapsing-remitting multiple sclerosis fulfilling study criteria. MS, multiple sclerosis; LP, lumbar puncture; RRMS, relapsing-remitting MS; IgM, immunoglobulin M; S, serum; CSF, cerebrospinal fluid.

This was a retrospective study including all relapsing-remitting multiple sclerosis (RRMS) patients who underwent a routine diagnostic MS investigation between 2001-2018 at the MS centre, Sahlgrenska University Hospital, Gothenburg, Sweden (**Figure 1**). All patients were registered in the Swedish MS registry (SMSreg, http://www.msreg.net) (32). Data about clinical relapses, follow-up MRIs, expanded disability status scale (EDSS) (33) and exposure to DMTs were collected from the SMSreg and patients’ electronic journals. All patients fulfilled the 2017 revised McDonald criteria at diagnosis (19). Only patients with determination of serum and CSF IgG and IgM, and cNfL were included. Samples were prospectively obtained and consecutively analysed by board-certified laboratory technicians in the Clinical Neurochemistry Laboratory at the Sahlgrenska University Hospital, Mölndal. The study population was limited to patients who had their diagnostic investigation within 12 weeks after their onset relapse. Additional inclusion criteria were an available MRI scan at baseline and a follow-up period of at least two years.

### Study endpoints

Three study endpoints were used for the purpose of our analyses. The first endpoint was evidence of disease activity 3 (EDA-3: clinical relapses; confirmed disability worsening (CDW) within 6 months (6-CDW), and new T1 gadolinium-enhanced lesions/new/newly enlarging T2-weighted [T2W] lesions) (34, 35) within a follow-up period of 24 months from diagnostic LP. A clinical relapse was defined as neurological signs and symptoms lasting at least 24 hours and that could not be explained by another cause (19). Brain and spinal cord MRI were performed on 1.5 and 3.0 T machines, according to Swedish radiological guidelines (36). Data on T1 gadolinium-enhanced lesions and new/newly enlarging T2W-lesions at follow-up was collected. CDW was defined as an increase in EDSS score from baseline sustained between two follow-up visits separated in time by no less than six months (1.5 point if EDSS at baseline was 0, 1 point if EDSS was between 1 and 5, 0.5 points if the baseline EDSS≥5.5). Patients were then dichotomized to those who maintained NEDA-3 status during the 24-month follow-up and those who experienced/showed evidence of disease activity (EDA-3). The second and third endpoints were EDSS≥3 and EDSS≥6 respectively. Thus, RRMS patients in our cohort were further dichotomized into those who had EDSS<3 and those that reached confirmed disability of EDSS≥3, and those with EDSS<6 and reached confirmed disability of EDSS≥6 along the total observational time including the last visit. In patients who did not reach the milestones of EDSS≥3 or EDSS≥6, disability was determined with EDSS at the last visit, provided that it was unchanged/not preceded by a recent relapse within the last six months.

### Serum and CSF analyses of IgM and IgG

Matched CSF and serum samples were obtained during routine diagnostic work-up and analysed consecutively. Levels of albumin and IgM in serum and CSF were measured on a cobas c module instrument (Roche) with the ALBT2, IGM-2 and IGM-C reagent cassettes. The albumin quotient (Q_Alb_) was calculated as CSF albumin (mg/L)/serum albumin (g/L) and the IgM-index was calculated as [(CSF IgM (mg/L)/serum IgM (g/L)) ÷ Q_Alb_]. IgM-index>0.1 was considered elevated (37, 38). Alternatively, the intrathecal fraction of IgM (IgM_IF_) was calculated according to the formula by Reiber (39). CSF-specific OCMB were determined by in-house agarose gel electrophoresis followed by an in-house immunoblotting method. Serum was diluted with saline solution to equal IgM-concentration as in the CSF sample. Agarose gel electrophoresis was performed using Hydragel 15HR kit (Sebia, product# 4122) on a Hydrasys 2 system (Sebia); paired CSF and diluted serum were analysed side by side on the gel. After electrophoresis the gel was briefly pre-blotted with filter paper. A polyvinylidene fluoride membrane (Immobilon-P pore size 0,45µm Millipore, cat# IPVH20200) was activated with ethanol, equilibrated in phosphate buffered saline (PBS) and then placed on the gel. Four filter papers wetted in PBS, a two cm layer of cellulose wadding and a two-kilogram weight was placed on the membrane and the gel was blotted for 60 minutes. The membrane was then blocked in PBS with Tween^®^20 0,1% for 15-30 minutes and then incubated with an alkaline phosphatase conjugated, polyclonal goat anti-human IgM antibody (Sigma cat# A3437) diluted in PBS with Tween^®^20 0,05% overnight. After four washes with PBS the blot was developed with SigmaFAST™ BCIP^®^/NBT (Sigma cat# B5655), the developed membrane was scanned while wet and then dried. Both the scanned image and the dry membrane were evaluated, and CSF-specific OCMB were defined as ≥2 IgM bands present in CSF which were not present in the matching serum sample. On each gel a CSF sample with known OCMB was included as quality control.

CSF- and serum IgG levels were analysed using the IGG-2 reagent cassette on a cobas c module instrument (Roche). The IgG-index was calculated as [(CSF IgG (mg/L)/serum IgG (g/L)) ÷ Q_Alb_]. IgG-index>0.7 was considered elevated. CSF-specific IgG OCBs were determined using an in-house IEF method on 7.7% polyacrylamide gels and subsequent silver staining. Paired patient serum and CSF samples were run on adjacent lanes, and CSF-specific IgG OCBs were defined as extra bands in the gamma-zone, which were not present in the corresponding serum sample. For quality control, a positive CSF sample with known CSF-specific OCBs was run on each gel. A cut-off of OCB≥2 was considered positive.

Board-certified laboratory technicians, who were blinded to the clinical status, using strict procedures for quality control and run-approval, performed the analyses.

### CSF analyses of NfL

cNfL was analyzed using a sensitive sandwich enzyme-linked immunosorbent assay (ELISA) method (NF-light^®^ ELISA kit; UmanDiagnostics AB, Umea, Sweden; catalogue # 10-7001 CE) and age-adjusted upper limits of the reference range utilized in clinical practice were used in order to determine whether cNfL levels were elevated or normal, as previously described (28). These upper limits are: <380 ng/L (<30 years), <560 ng/L (30-39 years), <890 ng/L (40-60 years), and <1850 ng/L (>60 years).

### Ethical considerations

All patients included in this study had given informed consent to be registered in the SMSreg. The study has been approved by the Swedish Ethical Review Agency (Dnr: 2019-01199).

### Statistics

Descriptive statistics are presented as median and interquartile ratio (IQR) or range (minimum-maximum). Categorical variables are described using relative and absolute frequencies. The measurement of agreement between the different methods to evaluate ITMS was determined with the kappa statistic.

To investigate the ability of ITMS to predict a worse disease course, the following endpoints were assessed: the association of ITMS with EDA-3, and the disability progression endpoints EDSS≥3 and EDSS≥6. For these endpoints, cox proportional hazards regression models were performed and the adjusted hazard ratios (aHR) along with corresponding 95% confidence intervals (CI) were calculated, as well as univariable Kaplan-Meier survival analyses with corresponding logrank tests. Kaplan-Meier curves are presented to visualize the results. For the EDA-3 endpoint, we adjusted for the following potential confounding factors: age at debut, sex, disease duration at diagnosis, T2W lesion burden at the time of diagnosis, as well as exposure to DMTs (first-line/second-line). In the case of EDA-3, the total follow-up time was 24 months. Patients who experienced EDA-3 during the follow-up period were censored at the time of the first signs of EDA-3. Those who fulfilled NEDA-3 at the end of the 24-month follow-up period were censored at 24 months. In the analyses of disability progression endpoints, patients were censored either at the time of reaching the investigated milestone or at the time of the last visit in case EDSS was kept <3 and <6 respectively. For the disability milestones, we adjusted for age at the time of diagnosis, sex, disease duration, baseline MRI T2W lesion burden, exposure to DMTs, and whether subjects escalated therapy during the follow-up. Time to EDA-3, EDSS≥3, and EDSS≥6 was analysed with Kaplan-Meier survival analysis and the logrank test. In order to investigate whether ITMS and cNfL have an additive predictive value, we then computed a new variable that combined positivity for IgM-index, IgM_IF_ or OCMB and cNfL, and performed cox proportional hazards regression with the same endpoints and adjustments as above. Due to the exploratory nature of the study, no adjustments for multiple comparisons were made.

The same analyses utilising cox proportional hazards models were performed independently and separately for IgG-index and cNfL. All statistical analyses were performed with IBM SPSS version 28.0.1.0 (Armonk, NY: IBM Corp. 2011). Figures were created in GraphPad prism version 9.1.0.

## Results

The study population included 457 RRMS patients, of which 316 (69%) were female, with a median (IQR) age at clinical onset of 35 (28–44). Males had significantly higher (median, IQR) IgM-index values (0.176, 0.09-0.312) compared with females (0.125, 0.08-0.218, p=0.004). Age did not influence ITMS. Median (IQR) follow-up time from lumbar puncture (LP) until the last follow-up visit was 7 years (4–12). CSF specific IgG-OCBs were present in 430 (94.1%) patients, in which 289 (97.3%) patients with IgM-index>0.1, 148 (98%) patients with IgM_IF_>0%, and 182 (96.7%) patients with OCMB had also OCGB. Demographic and clinical characteristics are presented in **Table 1**.

**Table 1:** Clinical and demographical characteristics of the study population.

	RRMS patients (n=457)
**Age, y, median (IQR)**	35 (28-44)
**Female, n (%)**	316 (69)
**Time from symptom onset to lumbar puncture, d, median (IQR)**	16 (6-34)
**Follow-up time from first to last visit, y, median (IQR)**	7 (4-12)
**EDSS at baseline, median (range)**	2 (0-7)
**EDSS at censoring/last-follow-up, median (range)**	2 (0-8)
**RRMS patients achieving EDSS milestones, n (%):** **EDSS≥3** **EDSS≥6**	136 (29.7%) 37 (8.1%)
**Number of MRI scans from baseline to censoring, median (IQR)**	3 (3-4)
**Median time to MRI scan, d, median (IQR)**	186 (119-259)
**T2 lesions at baseline, n (%)** • **1-9** • **10-20** • **>20**	193 (42.2) 111 (24.3) 153 (33.5)
**CSF specific IgG-OCB, n (%)**	430 (94.1)
**IgG-index>0.7, n (%)**	316 (69.1)
**CSF NFL (ng/L), median (IQR)**	832.5 (335-2074)
**DMT after first relapse, n (%)**	
**First-line:**	
Interferon-β Glatiramer-acetate Dimethyl-fumarate Teriflunomide	224 (49) 36 (7.9) 59 (12.9) 11 (2.4)
**Second-line**	
Natalizumab Fingolimod Rituximab Ocrelizumab Cladribine Alemtuzumab	65 (14.2) 12 (2.6) 12 (2.6) 5 (1.1) 1 (0.2) 4 (0.9)
**Switched to high efficacy DMT, n (%)**	200 (43.8%)

EDSS, expanded disability status scale; RRMS, relapsing remitting multiple sclerosis; CSF, cerebrospinal fluid; Ig, immunoglobulin; OCB, oligoclonal bands; DMT, disease modifying therapy.

### Intrathecal IgM synthesis

In 297 patients (65%), elevated IgM-index>0.1 was noted, whereas 151 (33%) patients had IgM_IF_>0%. The measurement of agreement between IgM-index>0.1 and IgM_IF_>0% according to the kappa statistic was ϰ=0.42 (p<0.001). Increasing the cut-off-value of IgM-index to ≥0.18 increased the measurement of agreement substantially to ϰ=0.87 (p<0.001). Likewise, 188 patients (44.7%) had OCMB, while 36 patients (7.9%) were missing a measurement of OCMB. The measurement of agreement between IgM-index and IgM_IF_≥0% with OCMB was fair (ϰ=0.3, p<0.001 and ϰ=0.33, p<0.001 respectively).

### NEDA-3

**Figure 2:**
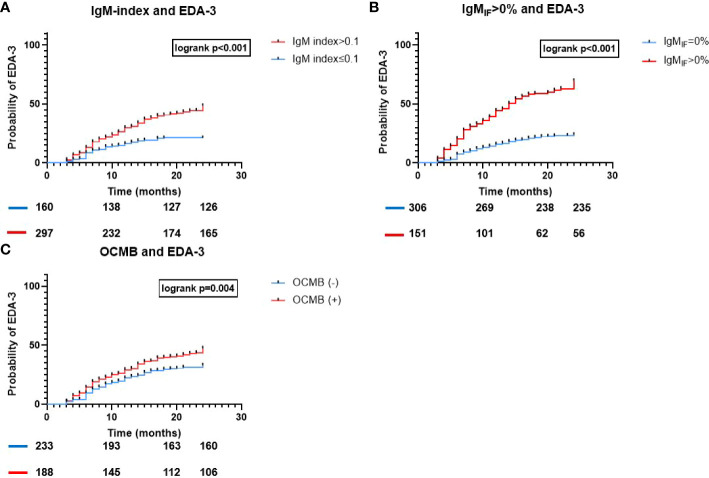
Time to EDA-3 for all IgM-metrics used to assess ITMS. Association of ITMS and time to EDA-3. Kaplan-Meier survival curves and results of the logrank test for **(A)** IgM-index>0.1; **(B)** IgM_IF_>0% according to Reiber; and **(C)** OCMB; and the probability of EDA-3 within 24 months from diagnostic LP; EDA, Evidence of Disease Activity; Ig, immunoglobulin; IF, intrathecal fraction; OCMB, oligoclonal IgM bands.

In our cohort, 178 patients (38.9%) did not fulfill NEDA-3 within 24 months. Both IgM-index and IgM_IF_ were moderately associated with a higher EDA-3 hazard (aHR 2.3, 95% CI 1.6-3.4, p<0.001; and aHR 3.7, 95%CI 2.7-5, p<0.001 respectively; **Table 2** and **Figure 2**), whereas OCMB showed a fair association with a higher hazard for EDA-3 (aHR 1.4, 95%CI 1.04-2, p=0.03). In a univariable analysis, the median (95%CI) time for attaining an EDA-3 status in patients with IgM-index>0.1, IgM_IF_≥0%, and OCMB was 15 (12.3-17.7), 14 (11.3-16.7), and 18 (16.8-19) months respectively. Patients with IgG-index>0.7 had an aHR of 1.6 (95%CI 1.1-2.3, p=0.006). Age was the only other covariate with a significant predictive effect in which rising age was slightly associated with a protective effect (aHR 0.96, 95%CI 0.95-0.98, p<0.001).

**Table 2:** Unadjusted and multivariable cox regression models for IgM metrics and prediction of 24-month EDA-3 status, EDSS≥3, and EDSS≥6.

	Univariable model			Cox proportional hazards		
	HR	95% CI	*p* value	aHR	95% CI	*p* value
EDA-3						
IgM-index>0.1	2.6	1.9-3.5	**<0.001**	2.3	1.6-3.4	**<0.001**
IgM_IF_>0%	3.9	2.8-5.5	**<0.001**	3.7	2.7-5	**<0.001**
OCMB	1.6	1.2-2.2	**0.004**	1.4	1.04-2	**0.03**
EDSS≥3						
IgM-index>0.1	1.6	1.1–2.3	**0.006**	1.9	1.3-2.8	**<0.001**
IgM_IF_>0%	1.1	0.8-1.6	0.5	1.4	0.9-2.1	0.06
OCMB	1.2	0.8-1.7	0.3	1.4	0.9-2.1	0.07
EDSS≥6						
IgM-index>0.1	1.6	0.8-2.9	0.2	2.1	1-4.4	0.05
IgM_IF_>0%	1.09	0.5-2.2	0.8	1.48	0.7-3	0.3
OCMB	1.9	1-3.9	0.05	2.5	1.2-5.4	**0.01**

HR, hazard ratio; aHR, adjusted hazard ratio; CI, confidence interval; EDA, Evidence of Disease Activity; Ig, immunoglobulin; IF, intrathecal fraction; OCMB, oligoclonal IgM bands; EDSS, expanded disability status scale.

Bold p values indicate statistical significance (p<0.05).

### Disability milestones

**Figure 3:**
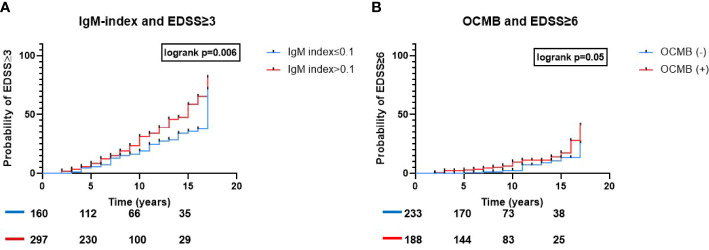
ITMS and risk of reaching disability milestones. Association of ITMS and risk of rising disability. Kaplan-Meier survival curves and results of the logrank test results for **(A)** IgM-index>0.1 and probability of reaching EDSS≥3 and **(B)** OCMB and probability of reaching EDSS≥6. Ig, immunoglobulin; EDSS, expanded disability status scale; OCMB, oligoclonal IgM bands.

EDSS≥3 was reached in 136 patients (29.8%) whereas 37 patients (8.1%) reached EDSS≥6 within a mean (range) follow-up period of 8.3 years (2-17). In a multivariable analysis, only patients with IgM-index>0.1 had a statistically significant higher risk to reach EDSS≥3 (aHR 1.9, 95%CI 1.3-2.8, p<0.001; **Table 2** and **Figure 3A**). In a univariable Kaplan-Meier analysis, the mean (95%CI) time to reach EDSS≥3 in patients with IgM-index>0.1 was 12.7 (12-13.4) years. However, when assessing EDSS≥6 as a milestone, IgM-index showed only a borderline significant higher risk (aHR 2.1, 95%CI 1-4.4, p=0.05; **Table 2**) whereas patients with OCMB showed significantly higher risk of reaching EDSS≥6 (aHR 2.5, 95% CI 1.2-5.4, p=0.01; **Table 2** and **Figure 3B**). Neither IgG-index>0.7 nor IgG-OCBs were significantly associated with progression to EDSS≥3 (aHR 1.4, 95%CI 0.9-2.1, p=0.08; aHR 1.7, 95%CI 0.6-4.7, p=0.3), nor with EDSS≥6 (aHR 0.6, 95%CI 0.3-1.2, p=0.13; aHR 0.8, 95%CI 0.2-3.6, p=0.8). Age but not gender, disease duration, T2 lesion-load or DMT exposure was associated with an increased risk of developing EDSS≥3 (aHR 1.04, 95%CI 1.03-1.06, p<0.001) and EDSS≥6 (aHR 1.06, 95%CI 1.03-1.09, p<0.001). In the analysis of IgM-index, switching therapy from a low efficacy DMT to a high efficacy DMT during the followup time was associated with progression to EDSS≥3 (aHR 1.8, 95%CI 1.2-2.8, p=0.01) but not with EDSS≥6.

### cNfL and prediction of EDA-3, EDSS≥3, and EDSS≥6

Age-adjusted elevated cNfL concentrations were associated with risk of EDA-3 status (aHR 1.5, 95%CI 1.1-2.1, p=0.02) as well as prediction of EDSS≥3 (aHR 2.5, 95%CI 1.7-3.6, p<0.001) and EDSS≥6 (aHR 3.1, 95%CI 1.5-6.4, p=0.003).

### ITMS and cNfL exhibit an additive predictive effect

**Figure 4:**
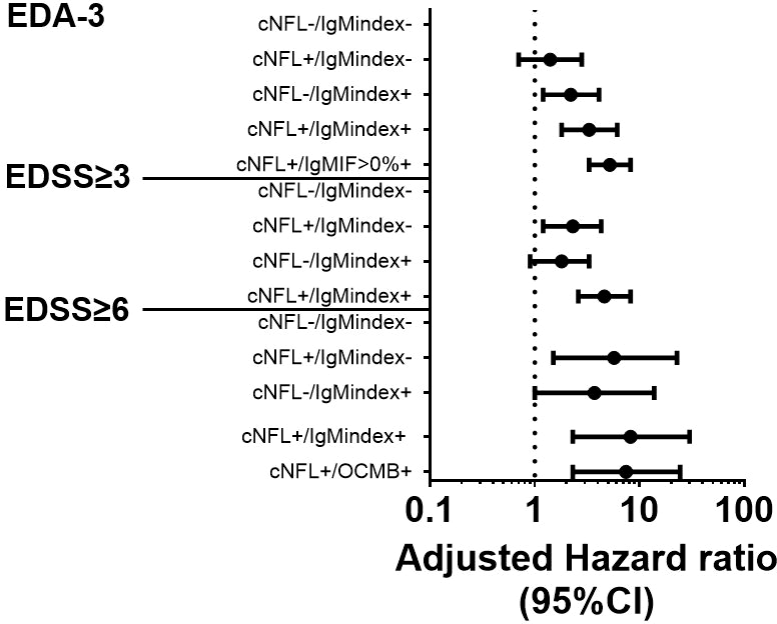
Combination of baseline cNFL and ITMS to predict EDA-3, EDSS≥3, and EDSS≥6. Forest plot with adjusted hazard ratios for risks of achieving EDA-3 status within 24 months, EDSS≥3, and EDSS≥6, stratified by combinatory possibilities of cNFL and ITMS. cNFL, cerebrospinal fluid neurofilament light; ITMS, intrathecal IgM synthesis; EDA, Evidence of Disease Activity; IgM, immunoglobulin M; IF, intrathecal fraction; OCMB, oligoclonal IgM band; EDSS, expanded disability status scale; Ig, immunoglobulin; CI, confidence interval.

Of the whole cohort, 77 patients (16.8%) were negative for both cNfL and IgM-index (cNfL-/IgM_(i)_-), 83 patients (18.2%) had elevated cNfL but IgM-index ≤ 0.1 (cNfL+/IgM_(i)_-), 174 patients (23.9%) had normal cNfL levels and IgM-index>0.1 (cNfL-/IgM_(i)_+), and 108 patients (38.1%) had elevated levels of both cNfL and IgM-index (cNfL+/IgM_(i)_+). As expected, in a multivariate analysis, patients with cNfL-/IgM_(i)_+ exhibited higher risk for EDA-3 (aHR 2.2, 95% CI 1.2-4.1, p=0.01; **Table 3** and **Figure 4**). Patients with cNfL+/IgM_(i)_+ showed an even higher risk for experiencing disease activity according to NEDA-3 (aHR 3.3, 95%CI 1.8-6.1, p<0.001; **Table 3** and **Figure 4**). When assessing ITMS with IgM_IF_>0%, patients with cNfL+/IgM_IF_+ showed the highest risk of EDA-3 (aHR 5.2, 95%CI 3.3-8.2, p<0.001, **Figure 4**).

**Table 3:** Combined cNFL and IgM-index.

	Cox proportional hazards		
	aHR	95% CI	*p* value
EDA-3			
cNFL-/IgMindex-	Ref.	–	–
cNFL+/IgMindex-	1.4	0.7-2.8	0.3
cNFL-/IgMindex+	2.2	1.2-4.1	**0.01**
cNFL+/IgMindex+	3.3	1.8-6.1	**<0.001**
cNFL-/IgM_IF_>0%-	Ref.	–	**-**
cNFL+/IgM_IF_>0%-	1.4	0.8-2.3	0.2
cNFL-/IgM_IF_>0%+	3.6	2.2-6	**<0.001**
cNFL+/IgM_IF_>0%+	4.9	3.1-7.8	**<0.001**
cNFL-/OCMB-	Ref.	–	–
cNFL+/OCMB-	2.1	1.2-3.6	**0.008**
cNFL-/OCMB+	1.8	0.9-3.3	0.06
cNFL+/OCMB+	2.8	1.6-4.7	**<0.001**
EDSS≥3			
cNFL-/IgMindex-	Ref.	–	–
cNFL+/IgMindex-	2.3	1.2-4.3	**0.01**
cNFL-/IgMindex+	1.8	0.9-3.3	0.06
cNFL+/IgMindex+	4.6	2.6-8.2	**<0.001**
cNFL-/IgM_IF_>0%-	Ref.	–	–
cNFL+/IgM_IF_>0%-	1.7	1.04-2.8	**0.035**
cNFL-/IgM_IF_>0%+	0.7	0.4-1.5	0.4
cNFL+/IgM_IF_>0%+	2	1.5-5.1	**<0.001**
cNFL-/OCMB-	Ref.	–	–
cNFL+/OCMB-	1.7	0.9-3.1	0.12
cNFL-/OCMB+	1.2	0.6-2.4	0.7
cNFL+/OCMB+	3.3	1.7-6.4	**<0.001**
EDSS≥6			
cNFL-/IgMindex-	Ref.	–	–
cNFL+/IgMindex-	5.7	1.5-22.7	**0.01**
cNFL-/IgMindex+	3.7	1-13.8	0.05
cNFL+/IgMindex+	8.2	2.3-30	**<0.001**
cNFL-/IgM_IF_>0%-	Ref.	–	–
cNFL+/IgM_IF_>0%-	2.4	0.9-6.2	0.06
cNFL-/IgM_IF_>0%+	0.8	0.2-2.9	0.8
cNFL+/IgM_IF_>0%+	3.4	1.05-10.9	**0.04**
cNFL-/OCMB-	Ref.	–	–
cNFL+/OCMB-	1.97	0.6-6.5	0.26
cNFL-/OCMB+	2	0.53-7.6	0.31
cNFL+/OCMB+	7.4	2.3-24.4	**<0.001**

aHR, adjusted hazard ratio; CI, confidence interval; EDA, Evidence of Disease Activity; cNFL, cerebrospinal fluid neurofilament light; Ig, immunoglobulin; IF, intrathecal fraction; EDSS, expanded disability status scale; OCMB, oligoclonal IgM bands. Multivariable analysis assessing the additive predictive value of cNFL and IgM-index regarding EDA-3 status at 24 months after the first relapse, EDSS≥3, and EDSS≥6. Bold p values indicate statistical significance (p<0.05).

Patients with elevated cNfL concentrations but IgM-index ≤ 0.1 had increased risk of developing EDSS≥3 and EDSS≥6 (aHR 2.3, 95%CI 1.2-4.3, p=0.01 and aHR 5.7, 95%CI 1.5-22.7, p=0.01 respectively; **Table 3** and **Figure 4**). However, patients with elevated cNfL concentrations and IgM-index>0.1 exhibited the highest risk of developing EDSS≥3 and EDSS≥6 (aHR 4.6, 95%CI 2.6-8.2, p<0.001 and aHR 8.2, 95%CI 2.3-30, p<0.001 respectively; **Table 3** and **Figure 4**). Since OCMB was the strongest predictor of EDSS≥6 in the first-step analysis, we computed a variable for cNfL/OCMB and investigated its predictive value for EDSS≥6. Ninety patients (21.4%) were cNfL-/OCMB-, 142 patients (33.8%) were cNfL+/OCMB-, 74 patients (17.6%) were cNfL-/OCMB+, and 114 patients (27.1%) were cNfL+/OCMB+. Only patients with cNfL+/OCMB+ showed a statistically significant result (aHR 7.4, 95%CI 2.3-24.4, p<0.001; **Table 3** and **Figure 4**).

## Discussion

In the present study, we investigated the prognostic value of ITMS, assessed with two quantitative methods and one qualitative method. Furthermore, we investigated the combination of ITMS and cNfL and examined whether analysis of these two prognostic biomarkers at diagnosis adds additional prognostic information. All of the data presented is retrieved from our clinical routine diagnostic investigations of RRMS during 17 years.

The agreement between IgM-index and IgM_IF_ was moderate, whereas it was fair between both quantitative methods and OCMB. These results are in line with previous reports (40). The discrepancy between the index and the Reiber formulas probably depends on the cut-off value for the index formula which is >0.1. When the cut-off value in our material is changed to ≥0.18, the measurement of agreement is almost perfect. However, since the cut-off >0.1 is the accepted cut-off for IgM-index in the literature, we chose to use it in our analyses. It is unclear why there is a discrepancy between the quantitative methods and the qualitative method. Qualitative assessment of ITMS is technically more challenging and therefore results might be less reliable.

We show that ITMS is a good predictor of future EDA-3 status within two years from diagnostic LP. In our analysis, IgM_IF_ and IgM-index were the strongest predictors of EDA-3 status, followed by OCMB. ITMS was also a good predictor of future disability worsening as determined by EDSS. Additionally, to the best of our knowledge, this is the first time that the combination of cNfL and ITMS at diagnosis is shown to demonstrate even stronger prognostic effect in predicting a more severe RRMS disease course.

Many of the patients previously classified as having CIS according to the 2010 McDonald criteria (41) are nowadays receiving an RRMS diagnosis earlier in the disease course, usually at the first demyelinating event, according to the revised 2017 McDonald criteria (19). In that sense, it is possible to compare CIS patients in previous studies who later converted to CDMS and those RRMS patients who present with a second relapse or more broadly, do not fulfil NEDA-3 criteria at follow-up. A large study recently found that the time to a first relapse was shorter and MS severity score (MSSS) higher in patients with IgM_IF_ (20). The same study showed that patients with IgM_IF_ had higher serum NfL, more new/enlarging T2W lesions, and higher total T2W lesion counts. Moreover, in patients with IgM_IF_≥median, the time to first relapse and to initiation of high-efficacy therapy was shorter in comparison to patients with IgM_IF_<median. In another recent work, Pfuhl et al. found that CIS patients with IgM_IF_>0% had a more than threefold risk of conversion to CDMS (12). Our results are thus similar and in line with these previous findings. In our investigation, IgM_IF_>0% and IgM-index were the strongest predictors of EDA-3, followed by OCMB, which nonetheless also showed significant results in both univariable and multivariable analyses.

ITMS has been previously shown to be associated with disability worsening as determined by EDSS (42, 43). However, several other studies were not able to confirm this association (14, 16, 17). Our data suggest that ITMS determined with IgM-index and OCMB, particularly in combination with cNfL is a strong predictor of clinical worsening over time. This combination of cNfL and ITMS seems to be particularly appealing, since both can be determined in CSF obtained from the diagnostic LP, and their synergistic predictive value seems to be strong for the risk of clinical worsening.

In a recent study, OCMB was associated with a 33% increase in the annualized relapse rate, higher odds for high-efficacy DMT, thinner peripapillary retinal nerve fibre layer (pRNFL), ganglion cell-inner plexiform layer (GCIPL), and higher rates of EDSS≥3 and EDSS≥4 (44). Furthermore, OCMB (9), and in particular lipid-specific (LS) OCMB (6, 13, 45), have been shown to be associated with a shorter time to first relapse. However, some studies were not able to confirm the prognostic value of OCMB (16).

LS-OCMB appears to be the strongest predictor of a second demyelinating event compared with other measures of ITMS (40) but this analysis is not available at our centre and is not used in clinical routine in most centres. A recent study compared IgM-index, IgM_IF_, OCMB, and LS-OCMB and investigated their ability to predict a second relapse in patients with CIS as well as achieving the disability milestones EDSS≥4, EDSS≥6, and conversion to SPMS (40). This study found that IgM-index was the poorest predictor of the above endpoints. However, in our investigation, quantitative estimations of ITMS performed best compared to OCMB when it comes to predicting future disease activity in a follow-up period of two years. In our analysis, IgM-index was a good predictor of EDSS≥3 and OCMB of EDSS≥6. We are therefore not convinced about the superiority of qualitative metrics over quantitative ones, especially keeping in mind the practical disadvantages involving IEF and immunoblotting. The predictive value of ITMS is nevertheless obvious, whether analysed quantitatively or qualitatively.

Notable limitations to our investigation are the retrospective nature of the study. This meant that the timing of MRI scans and clinical scoring was not completely harmonized between all research subjects. Some patients had their MRI scans before 3 Tesla MRI was widely available, and we did not have data on the number of scans performed with each magnetic field strength. Not all included patients had a qualitative analysis of OCMB which limits the comparison with the quantitative analyses. We were not able to determine LS-OCMB which might be an even stronger prognostic biomarker. However, our study material was consecutively collected as part of the diagnostic work-up in patients with suspected RRMS. The risk for selection bias in this real-world material was therefore low and the chosen endpoints are robust and clinically meaningful.

Recently, another approach to analyse the intrathecal fraction of Ig synthesis has been proposed by Auer et al. (46). The Auer formula has been shown to have less false positives compared to the Reiber formula, but in a recent study that compared all quantitative formulas to analyse IgM, results did not significantly differ in terms of prognostic value (47).

The reason why ITMS in comparison to ITGS shows both in our analysis as well as in previously published data (12, 20) a more convincing prognostic value is not completely known. Contrary to the process of immunoglobulin class switching commonly seen in peripheral tissues, in which initial production of IgM by B lymphocytes transitions into IgG production, intrathecal IgM secretion often persists in the CSF of MS patients, possibly due to high degree of somatic hypermutation in IgM-secreting CSF B lymphocytes (48). This could imply that IgM is involved in the pathogenesis of progression and influences degeneration, presumably due to the fact that OCMB often contain immunoglobulins directed against lipids that are found in abundance in myelin (22). Due to its pentameric structure, IgM effectively binds complement (49), leading to severe tissue injuries (50). Thus, hypothetically, the presence of ITMS in the immune-mediated attack on myelin and axons, may participate in disability worsening.

In our analysis, compared with ITMS, quantitative assessment of ITGS had weaker/no predictive value of disease severity in RRMS. This finding is in line with two previous investigations (20, 51) but contradicts a recently published work that showed an association between ITGS and disability worsening in MS (17). Reasons for these differences might be the higher prevalence of ITMS as well as a considerably longer follow-up time in our cohort, and the use of different statistical methods.

In conclusion, ITMS is a useful prognostic biomarker in early RRMS and we show for the first time that ITMS in combination with cNfL has an even stronger prognostic value. Our data suggest that determination of ITMS and cNfL should be incorporated in the diagnostic work-up of RRMS to extend the basis for prognosis and therapeutic decisions.

## Data availability statement

The raw data supporting the conclusions of this article will be made available by the authors, without undue reservation.

## Ethics statement

The studies involving human participants were reviewed and approved by Swedish Ethical Review Agency (Dnr: 2019-01199). Written informed consent for participation was not required for this study in accordance with the national legislation and the institutional requirements.

## Author contributions

IR and JL contributed to conception and design of the study. IR organized the database, performed the statistical analysis, and wrote the first draft of the manuscript. SR wrote sections of the manuscript. IR, MA, LN, KB, HZ, and JL had major role in data acquisition. All authors contributed to manuscript revision, read, and approved the submitted version.

## Funding

The study was supported by grants from the Swedish State Support for Clinical Research (ALFGBG-722081, ALFGBG-71320), Regional FoU grant Västra Götalandsregionen (260 101), NEURO Sweden, NEURO Gothenburg, Edith Jacobson’s Foundation and Helena Ahlin’s Foundation.

## Acknowledgments

KB is supported by the Swedish Research Council (#2017-00915), the Alzheimer Drug Discovery Foundation (ADDF), USA (#RDAPB-201809-2016615), the Swedish Alzheimer Foundation (#AF-930351, #AF-939721 and #AF-968270), Hjärnfonden, Sweden (#FO2017-0243 and #ALZ2022-0006), the Swedish state under the agreement between the Swedish government and the County Councils, the ALF-agreement (#ALFGBG-715986 and #ALFGBG-965240), the European Union Joint Program for Neurodegenerative Disorders (JPND2019-466-236), the National Institute of Health (NIH), USA, (grant #1R01AG068398-01), and the Alzheimer’s Association 2021 Zenith Award (ZEN-21-848495). HZ is a Wallenberg Scholar supported by grants from the Swedish Research Council (#2018-02532), the European Research Council (#681712 and #101053962), Swedish State Support for Clinical Research (#ALFGBG-71320), the Alzheimer Drug Discovery Foundation (ADDF), USA (#201809-2016862), the AD Strategic Fund and the Alzheimer’s Association (#ADSF-21-831376-C, #ADSF-21-831381-C and #ADSF-21-831377-C), the Olav Thon Foundation, the Erling-Persson Family Foundation, Stiftelsen för Gamla Tjänarinnor, Hjärnfonden, Sweden (#FO2019-0228), the European Union’s Horizon 2020 research and innovation programme under the Marie Skłodowska-Curie grant agreement No 860197 (MIRIADE), the European Union Joint Programme – Neurodegenerative Disease Research (JPND2021-00694), and the UK Dementia Research Institute at UCL (UKDRI-1003).

## Conflict of interest

IR has received compensation for lectures from biogen. MA has received compensation for lectures and/or advisory boards from Biogen, Genzyme, and Novartis. LN has received honoraria for lecture from Biogen, Novartis and Teva, and for advisory boards from Merck. KB has served as a consultant, at advisory boards, or at data monitoring committees for Abcam, Axon, BioArctic, Biogen, JOMDD/Shimadzu. Julius Clinical, Lilly, MagQu, Novartis, Ono Pharma, Roche Diagnostics, and Siemens Healthineers, and is a co-founder of Brain Biomarker Solutions in Gothenburg AB (BBS), which is a part of the GU Ventures Incubator Program, all unrelated to the work presented in this paper. HZ has served at scientific advisory boards and/or as a consultant for Abbvie, Alector, Annexon, Artery Therapeutics, AZTherapies, CogRx, Denali, Eisai, Nervgen, Novo Nordisk, Pinteon Therapeutics, Red Abbey Labs, Passage Bio, Roche, Samumed, Siemens Healthineers, Triplet Therapeutics, and Wave, has given lectures in symposia sponsored by Cellectricon, Fujirebio, Alzecure, Biogen, and Roche, and is a co-founder of Brain Biomarker Solutions in Gothenburg AB (BBS), which is a part of the GU Ventures Incubator Program (outside submitted work). JL has received travel support and/or lecture honoraria and has served on scientific advisory boards for Biogen, Novartis, and Sanofi Genzyme; and has received unconditional research grants from Biogen and Novartis.

The remaining author declares that the research was conducted in the absence of any commercial or financial relationships that could be construed as a potential conflict of interest.

## Publisher’s note

All claims expressed in this article are solely those of the authors and do not necessarily represent those of their affiliated organizations, or those of the publisher, the editors and the reviewers. Any product that may be evaluated in this article, or claim that may be made by its manufacturer, is not guaranteed or endorsed by the publisher.
